# Genetic interactions between Polycystin-1 and TAZ in osteoblasts define a novel mechanosensing mechanism regulating bone formation in mice

**DOI:** 10.21203/rs.3.rs-2957026/v1

**Published:** 2023-05-29

**Authors:** Zhousheng Xiao, Li Cao, Micholas Dean Smith, Hanxuan Li, Wei Li, Jeremy C. Smith, Leigh Darryl Quarles

**Affiliations:** 1 Department of Medicine, University of Tennessee Health Science Center, Memphis, TN, 38163; 2 Department of Pharmaceutical Sciences, University of Tennessee Health Science Center, Memphis, TN, 38163; 3 UT/ORNL Center for Molecular Biophysics, Oak Ridge National Laboratory, Oak Ridge, TN, 37830, USA.; 4 Department of Biochemistry and Cellular and Molecular Biology, The University of Tennessee-Knoxville, Knoxville, TN, 37996-1939

## Abstract

Molecular mechanisms transducing physical forces in the bone microenvironment to regulate bone mass are poorly understood. Here, we used mouse genetics, mechanical loading, and pharmacological approaches to test the possibility that polycystin-1 and TAZ have interdependent mechanosensing functions in osteoblasts. We created and compared the skeletal phenotypes of control *Pkd1*^flox/+^;*TAZ*^flox/+^, single *Pkd1*^*Oc-cKO*^, single *TAZ*^*Oc-cKO*^, and double *Pkd1/TAZ*^*Oc-cKO*^ mice to investigate genetic interactions. Consistent with an interaction between polycystins and TAZ in bone *in vivo*, double *Pkd1/TAZ*^*Oc-cKO*^ mice exhibited greater reductions of BMD and periosteal MAR than either single *TAZ*^*Oc-cKO*^ or *Pkd1*^*Oc-cKO*^ mice. Micro-CT 3D image analysis indicated that the reduction in bone mass was due to greater loss in both trabecular bone volume and cortical bone thickness in double *Pkd1*/*TAZ*^Oc-cKO^ mice compared to either single *Pkd1*^Oc-cKO^ or *TAZ*^Oc-cKO^ mice. Double *Pkd1*/*TAZ*^Oc-cKO^ mice also displayed additive reductions in mechanosensing and osteogenic gene expression profiles in bone compared to single *Pkd1*^Oc-cKO^ or *TAZ*^Oc-cKO^ mice. Moreover, we found that double *Pkd1/TAZ*^*Oc-cKO*^ mice exhibited impaired responses to tibia mechanical loading *in vivo* and attenuation of load-induced mechanosensing gene expression compared to control mice. Finally, control mice treated with a small molecule mechanomimetic MS2 had marked increases in femoral BMD and periosteal MAR compared to vehicle control. In contrast, double *Pkd1*/*TAZ*^Oc-cKO^ mice were resistant to the anabolic effects of MS2 that activates the polycystin signaling complex. These findings suggest that PC1 and TAZ form an anabolic mechanotransduction signaling complex that responds to mechanical loading and serve as a potential novel therapeutic target for treating osteoporosis.

## Introduction

*In vivo* and *in vitro* studies demonstrate that the polycystin-1(PC1)/polycystin-2 (PC2) heterotrimeric complex functions in osteoblasts and osteocytes to regulate bone mass ^1,2^ and acts as a mechanosensor to transduce the bone anabolic response to mechanical loading *in vivo* ^3,4^. Genetic ablation of either PC1 or PC2 deficiency in osteoblasts or osteocytes has similar effects in reducing bone mass by decreasing osteoblast-mediated bone formation ^3,5–8^. PC1 and PC2 mechanosensing functions are mediated by heterotrimeric complex activation of common signal transduction pathways. In this regard, PC1 and PC2 conditional knockout models exhibit concordant effects in impairing osteoblast-mediated bone formation. However, PC1 and PC2 have discordant effects on bone marrow adipogenesis that implicates separate signaling mechanism ^4,6,9^. In this regard, PC1 deficiency stimulates adipogenesis, leading to increased bone marrow adipose tissue (MAT) deposition ^2,6^, whereas PC2 loss-of-function inhibits adipogenesis^4^. Additional *in vitro* and *in vivo* data show that PC1 activates Runx2 transcriptional activity to stimulate osteoblastogenesis but diminishes PPARγ signaling leading to reduced bone marrow fat ^2,4^. In agreement with the low turnover bone disorder in *Pkd1* mouse models, the blood level of bone-specific alkaline phosphatase was significantly lower in patients with ADPKD compared to control subjects without ADPKD ^10–12^. The molecular mechanisms underlying the different effects of PC1 and PC2 on osteoblastogenesis and adipogenesis are not clear. In the current studies, we sought to understand the mechanism for the apparent PC1-specific effect in reciprocally regulating transcriptional control of osteoblastogenesis and adipogenesis.

The Hippo-YAP/TAZ pathway is also regulated by mechanical forces ^13–16^. Alterations of matrix stiffness in cell culture modulates nuclear translocation of non-phosphorylated TAZ resulting in co-activation of Runx2 and stimulation of osteoblastogenesis and in TAZ binding to PPARγ to inhibit adipogenesis ^2,17,18^. The physiological importance of TAZ in bone homeostasis is revealed by transgenic overexpression of *TAZ* in osteoblasts in mice, which increases osteoblast-mediated bone formation and inhibits bone marrow adipogenesis ^19^; depletion of *TAZ* in zebrafish, which impairs bone development ^18^, and global knockout of *TAZ* in mice, which have small stature and ossification defects ^20^. Based on these observations, we posit that PC1 dependent TAZ signaling might explain the differential functions of PC1 and PC2 on adipogenesis.

Recent studies show crosstalk between PC1 and TAZ signaling that is mediated by the binding of TAZ to the PC1 C-terminal tail ^2,17^. The PC1/TAZ complex is cleaved to allow nuclear translocation of TAZ, a mechanism of TAZ regulation that differs from the canonical Hippo regulation of YAP/TAZ signaling ^21^. TAZ binds to the PC1-CTT and undergoes nuclear translocation in response to changes in bone ECM microenvironment to stimulate osteoblastogenesis and inhibit adipogenesis through transcriptional co-activation of Runx2 and co-repression of PPARγ activity ^2^. TAZ also binds to PC2, leading to PC2 degradation^22^. These observations in bone parallel the interactions between PC1/PC2 and TAZ in primary cilia in renal epithelial cells^20,23,24^. In this regard, *TAZ* knockout from the kidney result in cystic kidney disease in mice, similar to polycystin complex inactivation, suggesting that PC1/PC2 and *TAZ* act through common pathways in the kidney as well as bone ^20,23,24^. Furthermore, a small molecule mechanomimetic (named as molecular staple, MS) that binds to the PC1:PC2 C-terminal tail in a presumptive coiled-coil region (e.g. PC1 residue Tyr^4236^ and PC2 residues Arg^877^, Arg^878^, and Lys^874^) has been shown to activate this complex and mimic the effects of physical forces to activate polycystins/TAZ signaling and stimulate bone mass in mice ^2^. Collectively these observations suggest that TAZ modulates polycystin’s mechanosensing functions to differentially regulate osteoblastogenesis and adipogenesis.

In this study, we examined the interaction between PC1 and TAZ in mouse bone by using *Osteocalcin* (*Oc*)-Cre to conditionally delete both *Pkd1* and *TAZ* in osteoblasts. We compared skeletal phenotypes of double *Pkd1/TAZ*^*Oc-cKO*^ mice with single conditional Pkd1 and TAZ null mice under baseline conditions, after mechanical loading, and following treatment with a more potent mechanomimetic, MS2, that activates the PC1/PC2 complex. We found that genetic ablation of PC1 and TAZ in osteoblasts results in additive loss of bone mass and anabolic responses to mechanical loading. Compound PC1 and TAZ deficient mice were also resistant to the bone inductive effects of the MS2 mechanomimetic *in vivo*. Our findings provide a new mechanism whereby TAZ regulates skeletal homeostasis through co-dependent functions with PC1 in osteoblasts.

## Results

### *TAZ* has gene-dosage dependent effects on bone mass.

**Fig 1.**
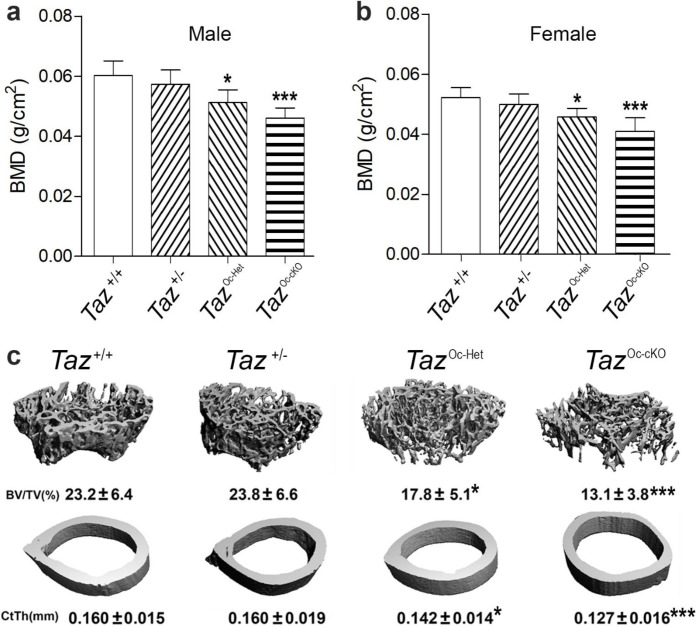
Conditional deletion of *TAZ* in mature osteoblasts on postnatal bone homeostasis. **a** & **b** Bone mineral density by DEXA scan in male and female mice. **c** Bone structure by micro-CT 3D images analysis from both male and female mice. Data are expressed as the mean ± S.D. from serum samples of individual mice (n=6). *P < 0.05, **P < 0.01, ***P < 0.001 compared with *wild-type* control mice. *P* values were determined by 1-way ANOVA with Tukey’s multiple-comparisons test.

**Fig 2.**
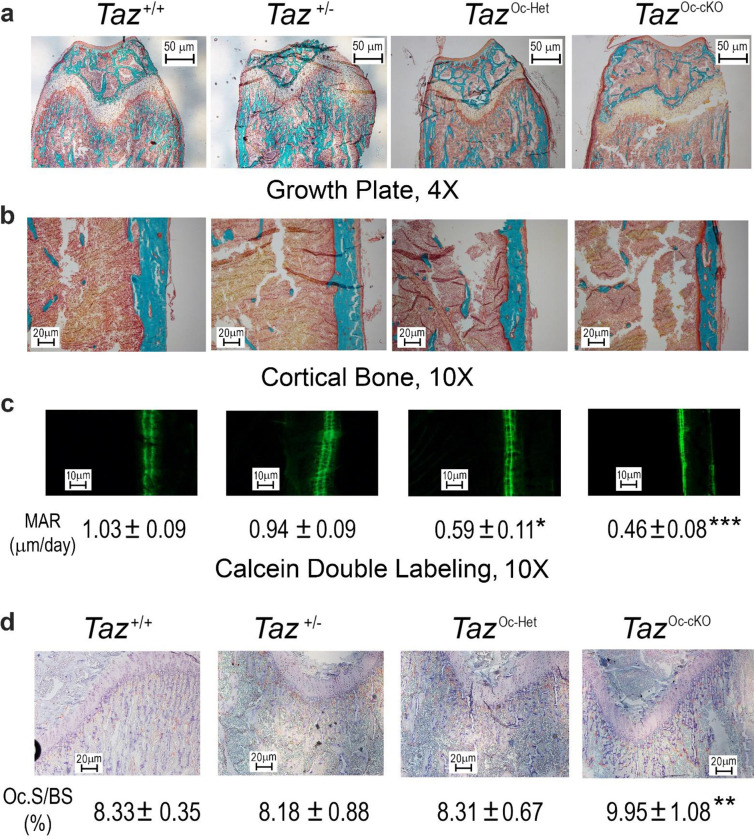
Histological analysis of conditional *TAZ* deletion in bone. **a** & **b** Goldner staining of non-decalcified bone. Representative images of distal femur sections displayed a gene-dosage dependent reduction in trabecular bone volume and cortical thickness from 8-week-old conditional *TAZ* deleted mice compared with age-matched control mice. **c** Periosteal mineral apposition rate (MAR) by calcein double labeling. There was a significant reduction in periosteal MAR in single *TAZ*^Oc-Het^ heterozygous mice compared with age-matched control mice and an even greater decrement in *TAZ*^Oc-cKO^ null mice, indicating a gene-dosage effect of TAZ on osteoblast-mediated bone formation. **d** TRAP staining (red color) for osteoclast activity. Data are expressed as the mean ± S.D. from 6 individual mice (n=6). *P < 0.05, **P < 0.01, ***P < 0.001 compared with *wild*-*type* control mice. *P* values were determined by 1-way ANOVA with Tukey’s multiple-comparisons test.

In the osteoblast specific conditional *TAZ* knockout mouse model, we found a gene-dosage effect on osteoblast-mediated bone formation and bone mass (Fig 1 & 2). Compared with control mice (*TAZ*^+/+^), heterozygous conditional *Oc*-Cre;*TAZ*^flox/+^ (*TAZ*^Oc-Het^), which has an approximately 37% reduction in *TAZ* message expression in bone (Table 1), showed 15% and 12% reductions of BMD in male and female adult mice, respectively (Fig 1A & 1B). *Oc*-Cre;*TAZ*^flox/−^ (*TAZ*^Oc-cKO^) mice, which had a 64% reduction in TAZ message expression in bone, showed an even greater loss of bone mass, with 24% and 22% reductions of BMD in male and female adult mice, respectively. Micro-CT 3D analysis showed that the reduction in bone mass in single conditional *TAZ*^Oc-Het^ heterozygous mice arose from a 23% reduction in trabecular bone volume and a 11% reduction in cortical bone thickness in both male and female adult mice. Conditional *TAZ*^Oc-cKO^ mice had a 44% loss in trabecular volume and 20% loss in cortical thickness.

In agreement with the DEXA and micro-CT data, analysis of bone histology showed a *TAZ* gene-dosage dependent reduction in trabecular bone volume (Fig 2A) and cortical bone thickness (Fig 2B) in the distal femur and a decrease in bone formation rate measured by double calcein labeling (Fig 2C). There were 43% and 55% reductions of periosteal MAR in both conditional *TAZ*^Oc-Het^ heterozygous and *TAZ*^Oc-cKO^ null mice, respectively, compared with control mice (*TAZ*^+/+^). Unexpectedly, conditional deletion of *TAZ* in osteoblasts resulted in enhanced osteoclast activity, as evidenced by increased TRAP immunostaining in the growth plate of conditional *TAZ*^Oc-cKO^ null mice (Fig 2D).

Real-time RT-PCR analysis revealed a *TAZ* gene-dosage dependent changes in osteoblast markers including *Runx2*-II and *Wnt10b, FGF-23*, *Mepe*, *RankL*, *CYR61*, and *CTGF* and chondrocyte markers such as *Collagen II* and *Collagen X.* Reductions of *Runx2-II* and *Wnt10b* impairs osteoblast proliferation and differentiation. Increments of *FGF-23* and *Mepe* as well as YAP signaling such as increased *CYR61* and *CTGF* gene expressions inhibits osteoblast differentiation and mineralization. An increase in *RankL* and the *RankL*/OPG ratio promotes osteoclast differentiation, leading to greater TRAP staining and higher osteoclast activity in conditional *TAZ*^Oc-cKO^ null mice compared with control mice (*TAZ*^+/+^). Conditional deletion of *TAZ* also resulted in increased adipocyte markers such as *PPARγ2*, *aP2* and *Lpl* gene expressions (Table 1).

Unexpectedly, global TAZ^+/−^ heterozygous mice, which had a ~40% reduction in TAZ message expression in bone, did not have significant changes in BMD or bone volume. Single-heterozygous *TAZ*^+/–^ showed normal bone gene expression profiles as well (Table 1). The osteoblast specific reduction but not global reductions of TAZ on the skeletal phenotype points to ether important co-factors in osteoblasts, such as TAZ interactions with PC1, or counteracting effects of TAZ in non-osteoblastic cells.

### Additive effects of combined *Pkd1* and *TAZ* deficiency to reduce bone mass.

**Fig 3.**
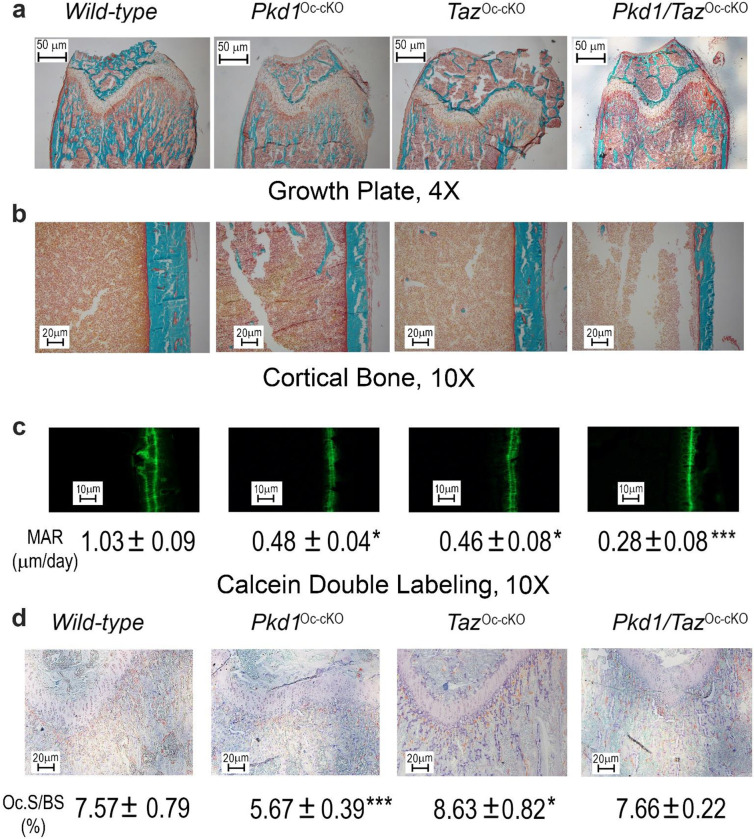
Conditional deletion of *Pkd1* and *TAZ* in mature osteoblasts on postnatal bone homeostasis. **a** & **b** Bone mineral density by DEXA scan in male and female mice. **c** Bone structure by micro-CT 3D images analysis from male mice. Data are expressed as the mean ± S.D. from serum samples of individual mice (n=6). *P < 0.05, **P < 0.01, ***P < 0.001 compared with *wild-type* mice, ^##^*P* < 0.01 compared with, *TAZ*^*Oc-cKO*^ mice, and ^&&^*P* < 0.01 compared with *Pkd1*^*Oc-cKO*^ mice, respectively. *P* values were determined by 1-way ANOVA with Tukey’s multiple-comparisons test.

**Fig 4.**
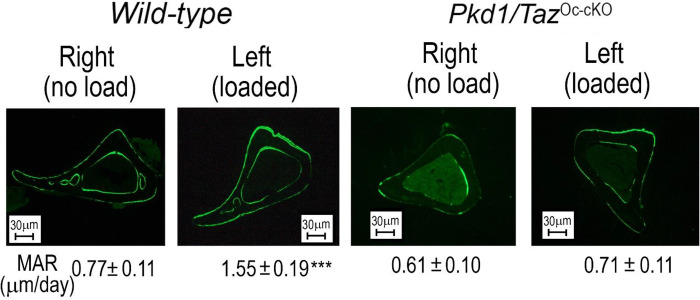
Histological analysis of conditional *Pkd1* and *TAZ* deletion in bone. **a** & **b** Goldner staining of non-decalcified bone. Representative images of distal femur sections displayed significant reductions in trabecular bone volume and cortical thickness from 8-week-old conditional *Pkd1* and/or *TAZ* deleted mice compared with age-matched control mice. **c** Periosteal mineral apposition rate (MAR) by Calcein double labeling. There was a significant reduction in periosteal MAR in single *Pkd1*^Oc-cKO^ or *TAZ*^Oc-cKO^ mice compared with age-matched control mice and an even greater decrement in double *Pkd1/TAZ*^*Oc-cKO*^ null mice, indicating a synergic effect of PC1 and TAZ on osteoblast-mediated bone formation. **d** TRAP staining (red color) for osteoclast activity. Data are expressed as the mean ± S.D. from 6 individual mice (n=6). *P < 0.05, **P < 0.01, ***P < 0.001 compared with *wild-type* mice. *P* values were determined by 1-way ANOVA with Tukey’s multiple-comparisons test.

To test the functional effects of the interaction between Pkd1 and TAZ in osteoblasts, we characterized *Osteocalcin*-Cre;*Pkd1*^flox/−^;*TAZ*^flox/−^ (*Pkd1*/*TAZ*^Oc-cKO^) double null mice with osteoblast-specific conditional deletions of both *Pkd1 and TAZ* in bone. In the conditional *Pkd1*/*TAZ* double knockout model, we found that *Pkd1* and *TAZ* have additive effects in reducing osteoblast-mediated bone formation. Compared with control mice (*Pkd1*^flox/+^*;TAZ*^flox/+^ ), both conditional *Pkd1*^Oc-cKO^ and *TAZ*^Oc-cKO^ null mice had similar reductions in bone mass, as evidenced by respective 22% and 21% reductions of BMD, 33% and 35% reductions in trabecular bone volume, and 18% and 19% reductions in cortical bone thickness as determined by distal femur Micro-CT 3D image analysis and Goldner staining (Fig 3 & 4). Also, the reductions in bone mass were similar in male and female adult mice.

The skeletal phenotype of double *Pkd1*/*TAZ*^Oc-cKO^ mice was more severe than either single *Pkd1*^Oc-cKO^ or *TAZ*^Oc-cKO^ null mice. Double *Pkd1*/*TAZ*^Oc-cKO^ mice had greater losses in BMD, trabecular bone volume, and cortical bone thickness with 33%, 53%, and 27% reductions, respectively in both male and female adult mice. This indicates the additive effects of Pkd1 and TAZ in postnatal bone homeostasis (Fig 3 & 4). Consistent with lower bone mass, combined *Pkd1* and *TAZ* deficiency also resulted in additive reductions in osteoblast- related gene expressions, such as in *Runx2-II*, *Osteocalcin*, and *Dmp1* (Table 2), as well as mechanosensing responsive genes such as in *Wnt10b, c-Jun, and PTGS2* (Table 2). Periosteal MAR (Fig 4) was decreased by ~73% in conditional double *Pkd1/TAZ*^*Oc-cKO*^ mice compared to controls, whereas *TAZ*^*Oc-cKO*^ and *Pkd1*^*Oc-cKO*^ single conditional knockout mice had reductions in periosteal MAR of 55% and 53%, respectively compared to control mice. Loss of either *Pkd1* or *TAZ* resulted in enhanced marrow adipogenesis, but no additive effects on adipocyte differentiation-related gene expressions were observed in the conditional double *Pkd1/TAZ*^*Oc-cKO*^ mice (Table 2).

We found that the conditional deletion of *Pkd1* or *TAZ* in osteoblasts has opposite effects on osteoclast activities. There was a decreased *RankL/OPG* expression ratio and TRAP immunostaining in *Pkd1*^*Oc-cKO*^ mice but an increased *RankL/OPG* expression ratio and TRAP immunostaining in *TAZ*^*Oc-cKO*^ mice (Table 2 and Fig 4). In contrast, double *Pkd1/TAZ*^*Oc-cKO*^ had similar *RankL* expression and TRAP immunostaining when compared to control, indicating a recovery of osteoclast activities in the double null mice (Table 2 and Fig 4). Changes in gene expression and immunostaining in bone correlated with alterations in serum biomarkers (Table 3). In this regard, further evidence for osteoblast and osteocyte dysfunction includes reductions in serum osteocalcin and FGF-23 from in single *Pkd1*^Oc-cKO^ or *TAZ*^Oc-cKO^ mice compared with age-matched control mice and an even greater decrement in double *Pkd1/TAZ*^*Oc-cKO*^ null mice (Table 3). In contrast, serum levels of TRAP, a marker of bone resorption, were decreased in single *Pkd1*^Oc-cKO^ mice, increased in single *TAZ*^Oc-cKO^ mice, but restored in double *Pkd1/TAZ*^*Oc-cKO*^ null mice compared with control littermates (Table 3). In addition, serum level of leptin was significantly higher in single *Pkd1*^Oc-cKO^ or *TAZ*^Oc-cKO^ mice than age-matched control mice. However, we did not observe further increase in double *Pkd1/TAZ*^*Oc-cKO*^ null mice (Table 3). These findings suggest that Pkd1 and TAZ have distinct functions among osteoblasts, adipocytes, and osteoclasts in bone *in vivo*.

### Loss of mechanical loading response in conditional *Pkd1* and *TAZ* deficient mice.

**Fig 5.**
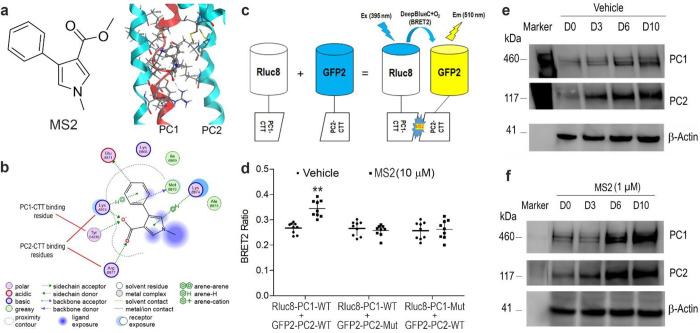
An impairment of anabolic response to mechanical loading in conditional *Pkd1* and *TAZ* deletion in bone. Representative images of midshaft tibia cross sections from no-load and loaded ulnae of *wild-type* control and compound *Pkd1/TAZ*^*Oc-cKO*^ null mice after loading. Data are mean ± S.D. from 6 tibias of *wild-type* control and *Pkd1/TAZ*^*Oc-cKO*^ mice. ***P < 0.001 compared with *wild-type* mice. *P* values were determined by 1-way ANOVA with Tukey’s multiple-comparisons test.

The cross-sections of tibiae from control and double *Pkd1/TAZ*^*Oc-cKO*^ null mice after mechanical tibia loading studies *in vivo* are shown in Fig 5. In *wild-type* control mice, loaded tibia showed a 2-fold increase in periosteal mineral apposition rate. In contrast, there was no measurable increase in periosteal mineral apposition in the loaded tibia from double *Pkd1*/*TAZ* knockout mice (Fig 5). In addition, a real-time RT-PCR analysis revealed that loaded tibia from the control mice had a dramatic response to mechanical stimulation, evidenced by significant increases of mechanosensing and osteogenic gene expressions including *Wnt10b*, *FzD2*, *Axin2*, *PTGS2*, *c-Jun*, *c-Fos*, *Runx2*-II, *Osteocalcin*, *ALP*, and *Dmp1* when compared with no load control tibia. In contrast, even when using the same loading regimen, no changes of mechanosensing and osteogenic gene expression profiles were observed in the loaded tibia from double *Pkd1/TAZ*^*Oc-cKO*^ null mice when compared with no load control tibia (Table 4). Thus, PC1 and TAZ are important in mediating mechanotransduction in bone.

### Validation of MS2 key binding to residues in PC1/PC2 C-terminus tails.

**Fig 6.**
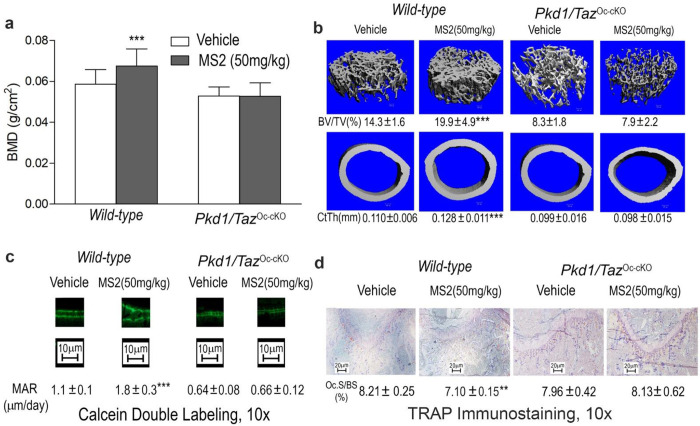
Cell-based BRET^2^ assays for MS2-target engagement assays. **a** Chemical structure of MS2 and an example of predicted 3D binding mode for MS2 (ball-and-sticks rendering in CPK colors) in PC2 (light blue). PC1 (red) as bound to PC2 in the homology structure is superimposed. **b** An example of calculated 2D binding mode and residues for MS2 in PC1/PC2 C-tails. **c** A diagram of BRET^2^ constructs and reactions in the presence of DeepBlue C with or without MS2 stimulation. **d** BRET^2^ signal changes from *wild-type* and mutant constructs with or without MS2 incubation. **e** & **f** Time-dependent changes of PC1 and PC2 proteins with or without MS2 incubation during osteogenic differentiation cultures in MC3T3-E1 cell line. Incubation Data are presented as the mean ± SD from 3 independent experiments (n = 3). **P < 0.01 compared with vehicle control.

We have previously showed that the small molecule MS2 activates PC1/PC2 complex signaling ^2^. Using computational modeling, we engaged in an induced fit docking campaign and predicted several potential ligand binding complexes. From these predicted poses, we identified key residues in the PC1 and PC2 C-terminus tail regions with which MS2 is predicted to interact. As shown in Fig 6, the PC2-CTT binding site for MS2 is predicted to include Lysine 874 and Arginine 877, whereas the PC1-CTT binding site for MS2 involves Tyrosine 4236 (Fig 6A & 6B). To test these predictions, we performed site-mutagenesis of key residues in both PC1 and PC2 and tested the effects of MS2 on PC1 and PC2 assembly using a BRET^2^ assay. We observed that the compound MS2 markedly enhances BRET^2^ signal in *wild-type* constructs, while mutagenesis of key residues in either PC1-CTT or PC2-CTT constructs completely abolished the BRET^2^ signal in the presence of compound MS2, confirming a role of MS2 in binding and enhancing the PC1 and PC2 interaction (Fig 6C & 6D).

Next, we examined PC1/PC2 complex formation during MC3T3-E1 osteoblast differentiation *in vitro*. We observed culture duration dependent increase of PC1/PC2 complex formation by western blot analysis. Incubation with 1 μM of MS2 in osteogenic cultures markedly increased the amount of PC1 and PC2 protein as assessed by western blot analysis (Fig 6E & 6F). These data suggests that MS2 may molecularly stabilize the PC1/PC2 complex in osteoblast culture *in vitro.*

### Loss of MS2-mediated stimulated increase in bone mass in conditional *Pkd1* and *TAZ* deficiency mice.

**Fig 7.**
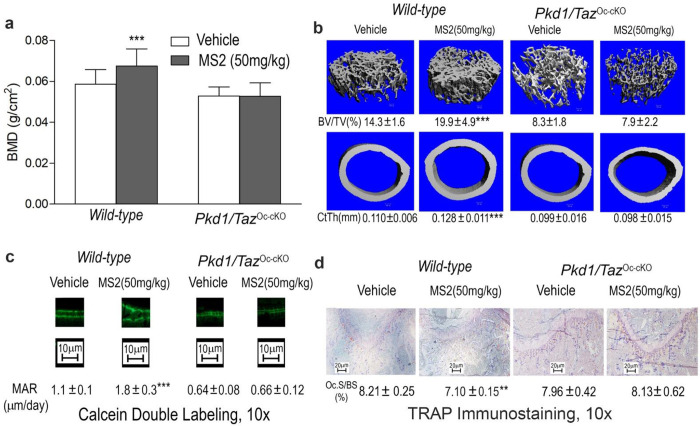
Effects of MS2 on bone formation in *wild-type* and compound *Pkd1/TAZ*^*Oc-cKO*^ null mice. **a** Bone mineral density by DEXA scan. **b** Bone structure by micro-CT 3D images analysis. **c** Periosteal mineral apposition rate (MAR) by Calcein double labeling. **d** Osteoclast activities by TRAP immunostaining after MS2 (50 mg/kg) treatment for 4 weeks compared to vehicle control. Data are mean ± S.D. from 6 tibias of *wild-type* control and compound *Pkd1/TAZ*^Oc-cKO^ null mice. *P < 0.05, **P < 0.01, ***P < 0.001 compared with *wild-type* control mice. *P* values were determined by 1-way ANOVA with Tukey’s multiple-comparisons test.

Finally, we treated *wild-type* and conditional double *Pkd1/TAZ*^*Oc-cKO*^ null mice with vehicle or MS2 (50 mg/kg) i.p. daily and assessed their skeletal response. After only 2 weeks, we observed that *wild-type* control mice treated with MS2 had a 15% increment in femoral bone mineral density compared to vehicle control (Fig 7). Micro-CT 3D images revealed that MS2 treated *wild-type* mice had a 39% increase in trabecular bone volume and 16% increase in cortical bone thickness.

In contrast, administration of MS2 had no effects on bone mineral density and bone structure in double *Pkd1/TAZ*^Oc-cKO^ null mice, suggesting specific-target dependent effects of MS2 on polycystins/TAZ signaling (Fig 7). We also observed that there were 1.6-fold increases in bone formation rate in *wild-type* mice treated with MS2 compared to the vehicle control, in agreement with enhanced osteoblastogenesis (e.g., *Runx2-II*, *OOsteocalcin, ALP* and *Dmp1*) and suppressed marrow adipogenesis (e.g., *PPARγ2*, *aP2,* and *Lpl*) by a real-time RT-PCR analysis (Table 5 and Fig 7). Again, administration of MS2 had no effects on bone formation rate and bone gene expression profiles in double *Pkd1/TAZ*^Oc-cKO^ null mice. Furthermore, MS2 stimulated mechanosensing gene expressions, including *Wnt1*, *Wnt10b*, *Axin-2*, *FzD2*, *c-Jun*, *c-Fos*, *eNOS*, and *PTGS2,* consistent with MS2 acting as a small molecule “mechanomimetic”. MS2 treatment decreased *RankL*/*OPG* expression ratio and TRAP immunostained osteoclasts in the MS2-treated mice compared to vehicle control (Table 5 and Fig 7). Administration of MS2 had no effects on osteoblast-mediated bone formation rate, marrow adipogenesis, and osteoclast activity in conditional double *Pkd1/TAZ*^*Oc-cKO*^ null mice (Table 5 and Fig 7). These data support that MS2 functions as anabolic drugs through the polycystins/TAZ pathway to promote the bone remodeling process.

## Discussion

In the current study, we provide loss-of-function genetic and gain-of-function pharmacological evidence for the co-dependent roles of PC1 and TAZ in regulating osteoblast-mediated bone formation and bone mass, First, using *Oc*-Cre to conditionally delete *Pkd1* and *TAZ* in osteoblasts in mice, we found that deletion of both genes in double *Pkd1/TAZ*^*Oc-cKO*^ null mice resulted in a more severe skeletal phenotype than loss of either PC1 or TAZ alone in the single *Pkd1*^*Oc-cKO*^ or *TAZ*^*Oc-cKO*^ null mice. Indeed, double *Pkd1*/*TAZ*^Oc-cKO^ null mice had greater reductions of bone mineral density and periosteal mineral apposition rate compared to single *TAZ*^*Oc-cKO*^ or *Pkd1*^*Oc-cKO*^ null mice. Micro-CT 3D image analysis revealed that this was due to greater loss in both trabecular bone volume and cortical bone thickness in double *Pkd1*/*TAZ*^Oc-cKO^ null mice compared to single *Pkd1*^Oc-cKO^ or *TAZ*^Oc-cKO^ null mice. Double *Pkd1*/*TAZ*^Oc-cKO^ null mice also exhibited greater reductions in osteoblast-related message expression in bone compared to single *Pkd1*^Oc-cKO^ or *TAZ*^Oc-cKO^ null mice.

Moreover, gene expression responses to tibia mechanical loading *in vivo* were also significantly impaired in double *Pkd1/TAZ*^*Oc-cKO*^ null mice, compared to control mice. These results are similar to our prior study showing that loss of *Pkd1* and *TAZ* had additive effects in decreasing bone mass in global *Pkd1* and *TAZ* double heterozygous mice ^2^. However, conditional *TAZ*^Oc-cKO^ mice had reductions in osteoblast-mediated bone formation whereas global or conditional *TAZ* heterozygous mice did not, suggesting the importance of the level and/or cell type expression of *TAZ* in determining the bone effects ^6^. Nevertheless, the current studies extend these findings by demonstrating that the bone phenotypes are due to direct loss of PC1/TAZ signaling in the osteoblast lineage. Moreover, these genetic loss-of-function studies indicate a functional interaction between PC1 and TAZ in bone *in vivo* under normal physiological conditions.

Second, consistent with our proposition that PC1/TAZ functions as a mechanosensor, double *Pkd1*/*TAZ*^Oc-cKO^ null mice in osteoblasts failed to respond to mechanical loading, similar to our previous report in the conditional deletion of *Pkd1* in osteocytes ^3^. The combined loss of PC1 and TAZ, however, resulted in greater reductions in mechanosensing responsive genes expressions in physiological conditions. The additive effects of the loss of TAZ and PC1 on mechanosensing responses can be explained by TAZ interactions with PC1 resulting in co-dependent signaling. Indeed, we confirmed that PC1-CTT interacts with PC2-CTT to produce BRET^2^ signal and that the small molecule MS2 enhances this interaction. Prior *in vitro* studies showed that overexpression of either full-length *PC1* or *PC1-CTT* along with full-length *PC2* constructs markedly increased TAZ-induced activation of TEAD activity and PC1 and TAZ form a complex and that cleavage of the PC1-C-Tail/TAZ complex by γ-secretase translocates to the nucleus to stimulate *Runx2- and TAZ*-mediated gene transcription, and this is the likely molecular mechanism explaining our observations ^2,17^. Our studies here did not explore alternative mechanisms for PC1 and TAZ functional interactions. In this regard, the TAZ/YAP Hippo pathway plays an essential role in mechanosensing of alterations in cell stiffness and extracellular matrix ^13–16^. Recent studies demonstrated that mechanical loading activates the TAZ/YAP pathway ^25–28^ and enhances TAZ/YAP nuclear translocation in response to shear stress in both bone marrow mesenchymal stem cells and MLO-Y4 osteocyte-like cell line ^29,30^.

Third, the importance of PC1/TAZ in regulating bone mass, suggest that this pathway is a potential target for pharmacological stimulation of bone formation. Indeed, we show here that treatment of mice with a small molecule, MS2, that targets the PC1/PC2/TAZ complex, has anabolic effects on bone, leading to stimulation of osteoblast mediated bone formation and increased bone mass. In this regard, administration of MS2 for up to 4 weeks in mice had significant increases in femoral BMD and periosteal MAR compared to the vehicle control. This response required PC1 and TAZ, since the administration of MS2 had no effects on femoral BMD and MAR in double *Pkd1*/*TAZ*^Oc-cKO^ null mice. The magnitude of the anabolic bone response to MS2 was similar to increments in bone mass in mice treated with PTH analogues and RANKL blocking antibodies that were developed to treat osteoporosis ^31^. These findings indicate that pharmacological activation of PC1/PC2 complex by MS2 promotes polycystin-1 and TAZ co-dependent anabolic effects on bone similar to preclinical studies of current treatments for osteoporosis. These results suggest that MS2 might be developed as a lead compound for treating osteoporosis by activating PC1/TAZ signaling.

Another interesting aspect of our studies is the observation that polycystin-1 and TAZ have opposite effects on osteoclast mediated bone resorption. Global *Pkd1* heterozygous mice showed fewer osteoclasts in bone as evidence by a lower number of TRAP positive osteoclasts ^2,3,6^, consistent with lower TRAP levels in ADPKD patients compared to non-ADPKD control ^10–12,32^. Conditional *Pkd1*^Oc-cKO^ null mice in osteoblast lineages with one global null and one conditional knockout alleles had greater reductions in osteoclast activities ^3,6^, indicating a gene-dosage dependent effect of loss-of-PC1 in osteoblasts impacting osteoclast functions, likely through paracrine effects ^33^. Whereas global *TAZ* heterozygous mice exhibited no changes in osteoclast activities compared to *wild-type* control mice ^2^, in our current study conditional *TAZ*^Oc-cKO^ null mice had higher osteoclast activities in bone as evidenced by increased number of TRAP positive osteoclasts. In contrast, double *Pkd1*/*TAZ*^Oc-cKO^ mice had normal osteoclast parameters, consistent with offsetting effects of conditional deletion of *Pkd1* and/or *TAZ* in osteoblasts on osteoclasts. These findings agree with previous publications regarding the effects of deletion of *Pkd1* ^2,3,6–8^ or *TAZ* ^25,34,35^.

Osteocytes regulate osteoclast activity through the RANKL/OPG paracrine pathway ^36,37^. PC1 and TAZ deficiency resulted in respective decrease and increase in *RANKL* expression but no difference in *OPG* expression in the *Pkd1*^Oc-cKO^ null and *TAZ*^*Oc-cKO*^ null mice, this could account for the differential effects of PC1 and TAZ on osteoclast activity in bone. Moreover, MS2 significantly decreased *RANKL* expression in bone and attenuated osteoclast activity. Our understanding of TAZ regulation of osteoclast function is further supported by the observation by Yang et al that either global or osteoclast-specific knockout of *TAZ* led to a low-bone mass phenotype due to elevated osteoclast formation ^38^. Thus, PC1 and TAZ signaling have divergent effects on osteoclasts and bone resorption.

Finally, a strong association exists in senile osteoporosis between decreased osteoblastogenesis and increased adipogenesis leading to increased bone marrow fat ^39–42^. We have previously reported that global PC1 deficiency in mice has an inverse effect, inhibiting osteoblastogenesis and stimulating adipogenesis ^2,7^. Similar to conditional *Pkd1*^Oc-cKO^ null mice ^3,6^, we also observed conditional *TAZ*^Oc-cKO^ null mice had greater increments in adipogenic markers than global or conditional *TAZ* heterozygous mice in the current study, suggesting a gene-dosage dependent effect of loss-of-TAZ in osteoblasts on bone marrow adipogenesis. Interestingly, we found a similar increase in adipogenic markers in both PC1 and/or TAZ osteoblast conditional knockout mice. The increase of adipogenic markers could be theoretically explained by increased transdifferentiation of osteoblast precursors to adipocytes ^43,44^, or effects of PC1 and TAZ in osteoblasts/osteocytes differentially releasing paracrine factors that modulate adipogenesis ^45–47^, analogous to paracrine factors that regulate osteoclastogenesis. Regardless, our studies revealed that double *Pkd1*/*TAZ*^Oc-cKO^ null mice had no differences in adipogenic markers relative to single *Pkd1*^Oc-cKO^ or *TAZ*^Oc-cKO^ null mice, suggesting that polycystin-1 and TAZ regulate adipocyte differentiation through the common polycystins/TAZ pathway.

In conclusion, polycystins and TAZ have interdependent effects in mediating bone mechanotransduction, regulating osteoblast mediated bone formation, and the anabolic skeletal responses to a small molecule mechanomimetic. These observations suggest that the PC1/PC2/TAZ complex is a novel drug target to treat age-related osteoporosis ^48,49^.

## Materials and methods

### Mice.

All animal studies were conducted according to guidelines provided by the National Institutes of Health and the Institute of Laboratory Animal Resources, National Research Council. The University of Tennessee Health Science Center’s Animal Care and Use Committee approved all animal studies (Protocol number: 21–0301). We obtained the floxed *TAZ* mice (*TAZ*^flox/flox^) which harbors two loxP sites flanking exon 2 of the *TAZ* gene from Drs. Jeff Wrana and Helen McNeill ^50^. We obtained the floxed *Pkd1* mice (*Pkd1*^flox/flox^) from Dr. Gregory Germino at Johns Hopkins University ^51^ and *Oc* (*Osteocalcin*)-Cre transgenic mice from Dr. Thomas Clemens at the University of Alabama ^52^. These mice were bred and maintained on a C57BL/6J background. At first, we used *Oc*-Cre;*TAZ*^+/−^ mice and homozygous *TAZ*^flox/flox^ mice to generate conditional *TAZ* heterozygous *Oc*-Cre;*TAZ*^flox/+^ (*TAZ*^Oc-Het^) and homozygous *Oc*-Cre;*TAZ*^flox/−^ (*TAZ*^Oc-cKO^) null mice as well as *TAZ* heterozygous mice (*TAZ*^+/−^) and Oc-Cre negative control mice (*TAZ*^flox/+^ equivalent to *wild-type*). Second, we also used *Oc*-Cre;*Pkd1*^+/−^ mice and homozygous *Pkd1*^flox/flox^ mice to generate *Oc*-Cre;*Pkd1*^flox/−^ (*Pkd1*^Oc-cKO^) mice. Then we used *Oc*-Cre;*Pkd1*^+/−^;*TAZ*^+/−^ mice and homozygous *Pkd1*^flox/flox^;*TAZ*^flox/flox^ mice to generate *Oc*-Cre;*Pkd1*^flox/−^;*TAZ*^flox/−^ (*Pkd1/TAZ*^Oc-cKO^) mice as previously described ^6,9^. These mice were used for skeletal phenotype analysis. The mice were anesthetized with Ketamine (90 mg/kg) and Xylazine (10 mg/kg) for LUNAR_PIXIMUS_ bone densitometer scan, and the mice not useful for experimental purposes were sacrificed by CO_2_ inhalation followed by cervical dislocation. In addition, we used wild-type C57BL/6J mice at 8 weeks of age to examine the effects of MS2 on osteogenesis and adipogenesis *in vivo*. The mice were treated with intraperitoneal injection of MS2 (50 mg/kg) i.p. or vehicle control (5% DMSO in PBS solution) once a day for 4 weeks. The bone samples were collected 4 hours after the last dose administration.

### Bone densitometry, histomorphometric and micro-CT analysis.

BMD of femurs was assessed at 8 weeks of age using a LUNAR_PIXIMUS_ bone densitometer (Lunar Corp, Madison, WI). Calcein (Sigma, St. Louise, MO) double labeling of bone and histomorphometric analyses of periosteal mineral apposition rate (MAR) in tibias and osteoclast surface per bone surface (Oc.S/BS, %) in femurs by TRAP immunostaining were performed using the osteomeasure analysis system (Osteometrics). The distal femoral metaphyses were also scanned using a Scanco μCT 40 (Scanco Medical AG, Brüttisellen, Switzerland). A 3D images analysis was done to determine bone volume (BV/TV) and cortical thickness (Ct.Th) as previously described ^3,4,6^.

### Real-time quantitative reverse transcription PCR (real-time qRT-PCR) and western blot analysis.

For real-time qRT-PCR, 1.0 μg total RNA isolated from either the long bone of 6-week-old mice or 8-days cultured BMSCs in differentiation media was reverse transcribed as previously described ^4,6^. PCR reactions contained 20 ng template (cDNA), 375 nM each forward and reverse primers, and 1 X EvaGreen Supermix (Bio-Rad, Hercules, CA) in 10 μl. The threshold cycle (Ct) of tested-gene product from the indicated genotype was normalized to the Ct for cyclophilin A. Then the tested-gene product vs cyclophilin A is normalized to the mean ratio of wild-type or control group, which has been set to 1.

For Western blot analysis, protein concentrations of the supernatant were determined with a total protein assay kit (Bio-Rad, Hercules, CA). Equal quantities of protein were subjected to 4–12% Bis-Tris or 3–8% Tris-Acetate gradient Gels (Invitrogen, Carlsbad, CA) and were analyzed with standard western blot protocols as previously described^4,6^. Polycystin-1 antibody (7E12, sc-130554), Polycystin-1 antibody (C-20, sc-10372), Polycystin-2 antibody (H-280, sc-25749), and Polycystin-2 antibody (YCE2, sc-47734) were purchased from Santa Cruz Biotechnology (Paso Robles, CA). Purified mouse TAZ antibody (560236) was purchased from BD Biosciences (San Jose, CA). Phosphorylated p-TAZ (Ser 89, sc-17610) and β-actin antibody (sc-47778) antibodies were from Santa Cruz Biotechnology (Paso Robles, CA). The intensity of the bands was quantified using Image J software (http://rsb.info.nih.gov/ij/).

### BRET^2^ assays for target engagement assay in vitro.

In collaboration with Oak Ridge National Laboratory and The University of Tennessee, Knoxville, we previously identified a compound that is thought to bind to the polycystin1 (PC1-CTT)/polycystin2 (PC2-CTT) complex in their C-terminus tails that we refer to as molecular staple two (MS2)^2^. Here, using computational ligand docking with an initial rigid receptor search using the proxy triangle algorithm and London dG scoring function and subsequent induced-fit refinement using a “free” receptor geometry, the Amber14SB force-field, and GBVI/WSA scoring function, as implemented in the Molecular Operating Environment (MOE, 2022) software package, against our previously identified binding pocket (in a previously published homology model of the PC1/PC2 CTT domain) we predicted over 30 different potential ligand-protein complexes. Investigation of these complexes identified two classes of binding poses, those dominated by PC2-ligand contacts and poses with PC1/PC2 bridging contacts. From the poses containing the largest number of ligand-protein contacts, we identified key residues of MS2 interacting with PC2-CTT [e.g., Arg(R)^877^ and Lys(K)^874^] and PC1-CTT [e.g., Tyr(Y)^4236^]. To confirm the interaction between MS2 and PC1/PC2 C-tail complex (1:3), we used PC1 C-terminal tail (PC1-CTT) and PC2 C-terminal tail (PC2-CTT) cDNAs to create Rluc8_PC1-CTT and GFP2_PC2-CTT constructs and develop BRET^2^ assays for their target engagement. The HEK-293T cells were transiently co-transfected with Rluc8_PC1-CTT (3.0 μg) and GFP2_PC2-CTT constructs (3.0 μg) by electroporation using a cell line optimal transfection kit according to the manufacturer’s protocol (Amaxa Inc, Gaithersburg, MD). The transfected cells were plated in 96-well black isoplate and cultured for 48 hours after transfection. We used the Synergy H4 plate reader to monitor the BRET^2^ signal (Fluorescence/Luminescence ratio). The relative fluorescence (515/30 nm) and luminescence (410/80 nm) raw data were detected from each well after adding DeepBlue C (5 μM) in the presence or absence of compound MS2 (10 μM). In addition, based on the identification of crucial contact residues [e.g. Lys(K)^874^ and Arg(R)^877^ in PC2-CTT and Tyr(Y)^4236^ in PC1] that bind to MS2 in the computational modeling, we used a Q5 site-directed mutagenesis kit to generate amino-acid residue substitutions at the interaction sites in *wild-type* GFP2_PC2-CTT (K874E & R877P) and Rluc8_PC1-CTT (Y4236F) cDNAs to create mutant constructs (GFP2_PC2-CTT- K874E & R877P and Rluc8_PC1-CTT-Y4236F mutants) that disrupt the contact sites in MS2-PC1/PC2 binding pocket of *wild-type* PC1-CTT/PC2-CTT complex. Then we used the same approach to co-transfect GFP2_PC2-CTT-K874E & R877P mutant along with *wild-type* Rluc8_PC1-CTT or Rluc8_PC1-CTT-Y4236F mutant along with *wild-type* GFP2_PC2-CTT constructs into HEK293 cells to measure the changes of the BRET^2^ signal after treated with vehicle or compound MS2 (10 μM).

### Synthesis of MS2.

A MS analogue, MS2 was synthesized to provide preliminary structure-activity relationship information based on the computational model shown in Figure 6A. The compound has a purity of greater than 98% and their structures were authenticated by standard analytical chemistry analyses.

### Statistical analysis.

We evaluated differences between two groups by unpaired t-test, multiple groups by one-way ANOVA with Turkey’s multiple comparison test. All values are expressed as means ± S.D. All computations were performed using a commercial biostatistics software (GraphPad Software Inc. La Jolla, CA).

## Figures and Tables

**Table 1.** Gene-expression profiles in bone from *TAZ*-deficient mice.

Gene	*Wild-type*	*TAZ^+/−^*	*TAZ^Oc-Het^*	*TAZ^Oc-cKO^*	*p*-value

**Osteoblast lineage**					
*Pkd1*	1.00±0.15	1.03±0.22	0.99±0.34	0.96±0.32	0.9718
*TAZ*	1.00±0.24	0.61±0.10***	0.63±0.14***	0.36±0.09***	<0.0001
*Fgf23*	1.00±0.10	0.97±0.22	1.61±0.45*	2.31±0.61***	<0.0001
*Runx2-II*	1.00±0.13	0.80±0.21	0.82±0.27	0.61±0.10**	0.0177
*Osteopontin*	1.00±0.26	0.83±0.20	0.67±0.11*	0.62±0.21*	0.0143
*Bsp*	1.00±0.11	0.94±0.28	0.68±0.16*	0.54±0.15***	0.0003
*Mepe*	1.00±0.12	1.04±0.22	1.43±0.28**	1.81±0.22***	<0.0001
*Col1*	1.00±0.20	0.81±0.23	0.67±0.21*	0.65±0.18*	0.0304
*Alp*	1.00±0.20	1.01±0.29	0.69±0.11*	0.52±0.15***	0.0002
*Osteocalcin*	1.00±0.19	0.80±0.23	0.53±0.19**	0.57±0.15**	0.0013
*OPG*	1.00±0.13	0.97±0.29	0.89±0.31	0.96±0.24	0.8926
*RankL*	1.00±0.17	1.13±0.24	1.12±0.11	1.52±0.15***	<0.0001
*Fzd2*	1.00±0.25	1.02±0.18	1.03±0.18	0.96±0.28	0.9354
*Wnt10b*	1.00±0.08	1.08±0.28	0.97±0.17	0.73±0.06*	0.0164
*CYR61*	1.00±0.13	1.06±0.10	1.54±0.25**	2.06±0.55***	<0.0001
*CTGF*	1.00±0.10	1.06±0.28	1.18±0.41	1.54±0.31*	0.0221
**Osteoclast**					
*Trap*	1.00±0.15	0.95±0.19	1.10±0.22	1.41±0.19**	0.0021
*Mmp9*	1.00±0.14	0.85±0.22	0.87±0.15	0.86±0.18	0.3808
**Chondrocyte**					
*Collagen II*	1.00±0.20	0.86±0.20	0.79±0.26	0.67±0.15*	0.0458
*Collagen X*	1.00±0.21	0.87±0.36	0.74± 0.39	0.45± 0.23*	0.0339
**Adipocyte**					
*PPARγ2*	1.00±0.17	1.23±0.14	1.57± 0.30**	2.03± 0.42***	<0.0001
*aP2*	1.00±0.18	1.27±0.31	1.64± 0.29**	2.51± 0.36***	<0.0001
*Lpl*	1.00±0.15	1.31±0.37	1.63± 0.34*	2.56± 0.47***	<0.0001

Data are mean ±S.D. from 6 tibias of *wild-type* control, *TAZ*^+/−^, *TAZ*^Oc-Het^, and *TAZ*^Oc-cKO^ mice and expressed as the fold changes relative to the housekeeping gene *β-actin* subsequently normalized to control mice.

*, **, *** indicates significant difference from *wild-type* control at *P*<0.05, *P*<0.01, *P*<0.001, respectively.

**Table 2.** Gene-expression profiles in bone from *Pkd1*^Oc-cKO^ or/and *TAZ*^Oc-cKO^ mice.

Gene	*Wild-type*	*Pkd1* ^Oc-cKO^	*TAZ* ^Oc-cKO^	*Pkd1/TAZ^Oc-cKO^*	*p*-value

**Osteoblast lineage**					
*Pkd1*	1.00±0.11	0.41±0.10***	0.93±0.31	0.45±0.10***	<0.0001
*TAZ*	1.00±0.24	0.98±0.27	0.46±0.10***	0.43±0.15***	<0.0001
*Pkd2*	1.00±0.12	1.05±0.21	0.93±0.25	1.09±0.31	0.6271
*Runx2-II*	1.00±0.14	0.76±0.11**	0.75±0.10*	0.62±0.21***	0.0017
*Osteocalcin*	1.00±0.13	0.75±0.15**	0.78±0.11**	0.66±0.11***	0.0010
*FGF-23*	1.00±0.13	0.55±0.17*	2.30±0.56***	1.02±0.25	<0.0001
*Dmp1*	1.00±0.20	0.80±0.10*	0.78±0.14*	0.65±0.10***	0.0012
*Wnt10b*	1.00±0.04	0.77±0.20**	0.73±0.19**	0.63±0.11***	0.0014
*Wnt1*	1.00±0.21	0.70±0.16*	0.92±0.35	0.52±0.21***	0.0007
*FzD2*	1.00±0.33	0.92±0.17	0.93±0.24	0.55±0.19**	0.0016
*c-Jun*	1.00±0.24	0.95±0.28	1.07±0.22	0.62±0.12**	0.0009
*c-Fos*	1.00±0.20	0.92±0.21	0.74±0.15*	0.67±0.13**	0.0011
*Axin-2*	1.00±0.31	0.77±0.10*	0.88±0.20	0.67±0.13***	0.0012
*PTGS2*	1.00±0.27	0.70±0.15*	0.68±0.16**	0.44±0.19***	<0.0001
*OPG*	1.00±0.19	0.93±0.26	1.08±0.26	0.97±0.18	0.7080
*RankL*	1.00±0.15	0.73±0.14*	1.45±0.13***	0.89±0.18	<0.0001
*CYR61*	1.00±0.13	1.40±0.18*	1.94±0.51***	1.67±0.33**	0.0005
*CTGF*	1.00±0.10	1.38±0.23*	1.68±0.30**	1.55±0.36**	0.0014
**Osteoclast**					
*Trap*	1.00±0.15	0.63±0.14**	1.43±0.20***	0.93±0.14	<0.0001
*Mmp9*	1.00±0.14	0.63±0.08*	0.86±0.18	0.95±0.35	0.0317
**Chondrocyte**					
*Collagen II*	1.00±0.19	0.92±0.24	0.73±0.15*	0.51±0.07***	<0.0001
*VegfA*	1.00±0.38	1.74±0.22*	1.66± 0.49*	1.70± 0.57*	0.0143
**Adipocyte**					
*PPARγ2*	1.00±0.20	1.78±0.34**	1.88± 0.51**	1.61± 0.22**	0.0010
*aP2*	1.00±0.16	1.71±0.33***	2.09± 0.31***	1.77± 0.38***	<0.0001
*Lpl*	1.00±0.13	1.62±0.28**	2.01± 0.51***	1.88± 0.38***	0.0004

Data are mean ± S.D. from 6 tibias of *wild-type* control, *Pkd1*^Oc-cKO^, *TAZ*^Oc-cKO^, and *Pkd1/TAZ*^*Oc*-cKO^ mice and expressed as the fold changes relative to the housekeeping gene *β-actin* subsequently normalized to control mice.

*, **, *** indicates significant difference from *wild-type* control at *P*<0.05, *P*<0.01, *P*<0.001.

**Table 3.** Biochemistry analysis of serum from *Pkd1*^Oc-cKO^ or/and *TAZ*^Oc-cKO^ mice.

Genotype	*Wild-type*	*Pkd1* ^Oc-cKO^	*TAZ* ^Oc-cKO^	*Pkd1/TAZ* ^Oc-cKO^	*p*-value

Urea nitrogen(mg/dl)	19 ± 5	22 ± 5	21 ± 6	22 ± 4	0.7236
Calcium (mg/dl)	10.3 ± 0.9	10.4 ± 1.3	10.2 ± 1.5	9.4 ± 0.9	0.3990
Phosphorus (mg/dl)	10.1 ± 1.9	10.4 ± 1.4	9.8 ± 2.4	10.3 ± 1.5	0.9355
Osteocalcin (ng/ml)	17.5 ± 3.5	3.4 ±0.7***	10.7 ± 2.6***	1.0 ± 0.8***	<0.0001
TRAP (units/liter)	10.8 ± 1.3	7.6 ±0.6***	12.2 ± 1.9*	9.8 ± 1.1	<0.0001
FGF23 (pg/ml)	117 ± 32	79 ± 16*	116 ± 35	46 ± 16***	0.0704
Leptin (pg/ml)	836 ± 200	2636 ± 759***	2649 ± 915***	1793 ± 600*	<0.0001

Data are mean ± S.D. from 6 serum samples of *wild-type* control, *Pkd1*^Oc-cKO^, *TAZ*^Oc-cKO^, and *Pkd1/TAZ*^Oc-cKO^ mice

*, **, *** indicates significant difference from *wild-type* control at *P*<0.05, *P*<0.01, *P*<0.001.

**Table 4.** Mechanosensing gene-expression profiles that respond to mechanical loading in *wild-type* and double *Pkd1/TAZ*^Oc-cKO^ mice.

Gene	*Wild-type* (no load)	*Wild-type* (loaded)	*Pkd1/TAZ*^Oc-cKO^ (no load)	*Pkd1/TAZ*^Oc-cKO^ (loaded)

*Wnt10b*	1.00±0.32	1.74±0.62**	1.00±0.31	0.94±0.31
*c-Jun*	1.00±0.27	1.72±0.41***	1.00±0.18	0.95±0.29
*c-Fos*	1.00±0.22	2.04±0.66***	1.00±0.24	0.92±0.29
*Axin-2*	1.00±0.16	1.54±0.25***	1.00±0.19	0.92±0.33
*PTGS2*	1.00±0.16	3.34±1.05***	1.00±0.22	1.15±0.17
*Runx2-II*	1.00±0.28	1.94±0.54***	1.00±0.19	0.92±0.19
*FzD2*	1.00±0.26	1.92±0.47***	1.00±0.26	0.93±0.32

Data are mean ± S.D. from 6 tibiae of *wild-type* control and *Pkd1/TAZ*^Oc-cKO^ mice and expressed as the fold changes relative to the housekeeping gene *eEF1a1* subsequently normalized to control or *Pkd1/TAZ*^Oc-cKO^ no load tibiae.

*, **, *** indicates significant difference from *wild-type* or *Pkd1/TAZ*^Oc-cKO^ no load control at *P*<0.05, *P*<0.01, *P*<0.001.

**Table 5.** Gene expression profiles in bone from MS2-treated *wild-type* control and *Pkd1/TAZ*^Oc-cKO^ mice.

Gene	*Wild-type* (Vehicle)	*Wild-type* (MS2)	*Pkd1/TAZ^Oc-cKO^* (Vehicle)	*Pkd1/TAZ^Oc-cKO^* (MS2)

*Pkd1*	1.00±0.13	0.94±0.15	1.00±0.37	1.03±0.28
*TAZ*	1.00±0.32	1.01±0.32	1.00±0.40	0.82±0.19
*Runx2-II*	1.00±0.19	1.79±0.52**	1.00±0.32	1.03±0.28
*Osteocalcin*	1.00±0.24	1.68±0.24***	1.00±0.57	0.86±0.61
*ALP*	1.00±0.14	1.56±0.23***	1.00±0.52	1.09±0.55
*Dmp1*	1.00±0.18	1.45±0.17***	1.00±0.33	1.02±0.48
*OPG*	1.00±0.38	1.10±0.37	1.00±0.30	1.18±0.33
*RankL*	1.00±0.30	0.57±0.15*	1.00±0.50	0.99±0.33
*Trap*	1.00±0.26	0.56±0.10*	1.00±0.58	0.93±0.28
*PPARγ2*	1.00±0.43	0.59±0.11*	1.00±0.47	1.06±0.18
*aP2*	1.00±0.22	0.60±0.26*	1.00±0.22	1.07±0.12
*Lpl*	1.00±0.28	0.63±0.12*	1.00±0.40	0.95±0.39
*Wnt1*	1.00±0.30	3.02±0.98***	1.00±0.60	0.97±0.40
*Wnt10b*	1.00±0.15	1.52±0.25***	1.00±0.40	1.12±0.21
*FzD2*	1.00±0.26	3.36±1.56***	1.00±0.30	0.95±0.18
*c-Fos*	1.00±0.20	1.47±0.34*	1.00±0.22	1.00±0.27
*c-Jun*	1.00±0.23	1.74±0.24***	1.00±0.29	0.84±0.20
*Axin-2*	1.00±0.22	2.57±1.02**	1.00±0.16	0.92±0.17
*PTGS2*	1.00±0.35	2.25±0.78**	1.00±0.26	0.90±0.22
*eNOS*	1.00±0.45	3.09±0.68***	1.00±0.13	1.03±0.22

Data are mean ± S.D. from 6 tibias of *wild-type* control and *Pkd1/TAZ*^Oc-cKO^ mice and expressed as the fold changes relative to the housekeeping gene *eEF1a1* subsequently normalized to control or *Pkd1/TAZ*^Oc-cKO^ no load tibias.

*, **, *** indicates significant difference from *wild-type* control at *P*<0.05, *P*<0.01, *P*<0.001.

## References

[1] Qin L., Liu W., Cao H. & Xiao G. Molecular mechanosensors in osteocytes. Bone Res 8, 23 (2020). 10.1038/s41413-020-0099-y 32550039 PMC7280204

[2] Xiao Z. Polycystin-1 interacts with TAZ to stimulate osteoblastogenesis and inhibit adipogenesis. J Clin Invest 128, 157–174 (2018). 10.1172/JCI93725 29202470 PMC5749530

[3] Xiao Z. Conditional deletion of Pkd1 in osteocytes disrupts skeletal mechanosensing in mice. FASEB J 25, 2418–2432 (2011). 10.1096/fj.10-180299 21454365 PMC3219213

[4] Xiao Z. Osteoblast-specific deletion of Pkd2 leads to low-turnover osteopenia and reduced bone marrow adiposity. PLoS One 9, e114198 (2014). 10.1371/journal.pone.0114198 25464512 PMC4252138

[5] Qiu N., Xiao Z., Cao L., David V. & Quarles L. D. Conditional mesenchymal disruption of pkd1 results in osteopenia and polycystic kidney disease. PLoS One 7, e46038 (2012). 10.1371/journal.pone.0046038 23029375 PMC3448720

[6] Xiao Z. Conditional disruption of Pkd1 in osteoblasts results in osteopenia due to direct impairment of bone formation. J Biol Chem 285, 1177–1187 (2010). 10.1074/jbc.M109.050906 19887454 PMC2801246

[7] Xiao Z., Zhang S., Magenheimer B. S., Luo J. & Quarles L. D. Polycystin-1 regulates skeletogenesis through stimulation of the osteoblast-specific transcription factor Runx2-II. J Biol Chem (2008). 10.1074/jbc.M710407200 PMC2335361 18321855

[8] Xiao Z. Cilia-like structures and polycystin-1 in osteoblasts/osteocytes and associated abnormalities in skeletogenesis and Runx2 expression. J Biol Chem 281, 30884–30895 (2006). 16905538 10.1074/jbc.M604772200 PMC1797154

[9] Qiu N., Cao L., David V., Quarles L. D. & Xiao Z. Kif3a deficiency reverses the skeletal abnormalities in Pkd1 deficient mice by restoring the balance between osteogenesis and adipogenesis. PLoS One 5, e15240 (2010). 10.1371/journal.pone.0015240 21151991 PMC2996304

[10] Evenepoel P. A distinct bone phenotype in ADPKD patients with end-stage renal disease. Kidney Int 95, 412–419 (2019). 10.1016/j.kint.2018.09.018 30665572

[11] Gitomer B. Mineral bone disease in autosomal dominant polycystic kidney disease. Kidney Int 99, 977–985 (2021). 10.1016/j.kint.2020.07.041 32926884 PMC7993988

[12] De Rechter S. Evidence for Bone and Mineral Metabolism Alterations in Children With Autosomal Dominant Polycystic Kidney Disease. J Clin Endocrinol Metab 102, 4210–4217 (2017). 10.1210/jc.2017-01157 29092060

[13] Dupont S. Role of YAP/TAZ in mechanotransduction. Nature 474, 179–183 (2011). 10.1038/nature10137 21654799

[14] Aragona M. A mechanical checkpoint controls multicellular growth through YAP/TAZ regulation by actin-processing factors. Cell 154, 1047–1059 (2013). 10.1016/j.cell.2013.07.042 23954413

[15] Dupont S. Role of YAP/TAZ in cell-matrix adhesion-mediated signalling and mechanotransduction. Exp Cell Res 343, 42–53 (2016). 10.1016/j.yexcr.2015.10.034 26524510

[16] Panciera T., Azzolin L., Cordenonsi M. & Piccolo S. Mechanobiology of YAP and TAZ in physiology and disease. Nat Rev Mol Cell Biol 18, 758–770 (2017). 10.1038/nrm.2017.87 28951564 PMC6192510

[17] Merrick D. Polycystin-1 regulates bone development through an interaction with the transcriptional coactivator TAZ. Hum Mol Genet 28, 16–30 (2019). 10.1093/hmg/ddy322 30215740 PMC6298236

[18] Hong J. H. TAZ, a transcriptional modulator of mesenchymal stem cell differentiation. Science 309, 1074–1078 (2005). 10.1126/science.1110955 16099986

[19] Yang J. Y. Osteoblast-targeted overexpression of TAZ increases bone mass in vivo. PLoS One 8, e56585 (2013). 10.1371/journal.pone.0056585 23441207 PMC3575506

[20] Hossain Z. Glomerulocystic kidney disease in mice with a targeted inactivation of Wwtr1. Proceedings of the National Academy of Sciences of the United States of America 104, 1631–1636 (2007). 10.1073/pnas.0605266104 17251353 PMC1785239

[21] Varelas X. The Hippo pathway effectors TAZ and YAP in development, homeostasis and disease. Development 141, 1614–1626 (2014). 10.1242/dev.102376 24715453

[22] Tian Y. TAZ promotes PC2 degradation through a SCFbeta-Trcp E3 ligase complex. Mol Cell Biol 27, 6383–6395 (2007). 10.1128/MCB.00254-07 17636028 PMC2099608

[23] Piontek K., Menezes L. F., Garcia-Gonzalez M. A., Huso D. L. & Germino G. G. A critical developmental switch defines the kinetics of kidney cyst formation after loss of Pkd1. Nature medicine 13, 1490–1495 (2007). 10.1038/nm1675 PMC2302790 17965720

[24] Lin F. Kidney-specific inactivation of the KIF3A subunit of kinesin-II inhibits renal ciliogenesis and produces polycystic kidney disease. Proc Natl Acad Sci U S A 100, 5286–5291 (2003). 12672950 10.1073/pnas.0836980100 PMC154337

[25] Kegelman C. D., Collins J. M., Nijsure M. P., Eastburn E. A. & Boerckel J. D. Gone Caving: Roles of the Transcriptional Regulators YAP and TAZ in Skeletal Development. Curr Osteoporos Rep 18, 526–540 (2020). 10.1007/s11914-020-00605-3 32712794 PMC8040027

[26] Zarka M. Mechanical loading activates the YAP/TAZ pathway and chemokine expression in the MLO-Y4 osteocyte-like cell line. Lab Invest 101, 1597–1604 (2021). 10.1038/s41374-021-00668-5 34521992

[27] Chen Z., Luo Q., Lin C. & Song G. Simulated microgravity inhibits osteogenic differentiation of mesenchymal stem cells through down regulating the transcriptional co-activator TAZ. Biochem Biophys Res Commun 468, 21–26 (2015). 10.1016/j.bbrc.2015.11.006 26549225

[28] Chen Z., Luo Q., Lin C., Kuang D. & Song G. Simulated microgravity inhibits osteogenic differentiation of mesenchymal stem cells via depolymerizing F-actin to impede TAZ nuclear translocation. Sci Rep 6, 30322 (2016). 10.1038/srep30322 27444891 PMC4957213

[29] Li X. Stimulation of Piezo1 by mechanical signals promotes bone anabolism. Elife 8 (2019). 10.7554/eLife.49631 PMC6779475 31588901

[30] Kim K. M. Shear stress induced by an interstitial level of slow flow increases the osteogenic differentiation of mesenchymal stem cells through TAZ activation. PLoS One 9, e92427 (2014). 10.1371/journal.pone.0092427 24658423 PMC3962409

[31] Furuya Y. Increased bone mass in mice after single injection of anti-receptor activator of nuclear factor-kappaB ligand-neutralizing antibody: evidence for bone anabolic effect of parathyroid hormone in mice with few osteoclasts. The Journal of biological chemistry 286, 37023–37031 (2011). 10.1074/jbc.M111.246280 21862583 PMC3196100

[32] Zubidat D. Bone health in autosomal dominant polycystic kidney disease (ADPKD) patients after kidney transplantation. Bone Rep 18, 101655 (2023). 10.1016/j.bonr.2023.101655 36659900 PMC9842864

[33] Kim J. M., Lin C., Stavre Z., Greenblatt M. B. & Shim J. H. Osteoblast-Osteoclast Communication and Bone Homeostasis. Cells 9 (2020). 10.3390/cells9092073 PMC7564526 32927921

[34] Kegelman C. D. Skeletal cell YAP and TAZ combinatorially promote bone development. FASEB J 32, 2706–2721 (2018). 10.1096/fj.201700872R 29401582 PMC5901392

[35] Xiong J., Almeida M. & O’Brien C. A. The YAP/TAZ transcriptional co-activators have opposing effects at different stages of osteoblast differentiation. Bone 112, 1–9 (2018). 10.1016/j.bone.2018.04.001 29626544 PMC5970058

[36] Xiong J. & O’Brien C. A. Osteocyte RANKL: new insights into the control of bone remodeling. J Bone Miner Res 27, 499–505 (2012). 10.1002/jbmr.1547 22354849 PMC3449092

[37] Kim H. N. Osteocyte RANKL is required for cortical bone loss with age and is induced by senescence. JCI Insight 5 (2020). 10.1172/jci.insight.138815 PMC7566701 32870816

[38] Yang W. TAZ inhibits osteoclastogenesis by attenuating TAK1/NF-kappaB signaling. Bone Res 9, 33 (2021). 10.1038/s41413-021-00151-3 34253712 PMC8275679

[39] Yeung D. K. Osteoporosis is associated with increased marrow fat content and decreased marrow fat unsaturation: a proton MR spectroscopy study. J Magn Reson Imaging 22, 279–285 (2005). 10.1002/jmri.20367 16028245

[40] Suchacki K. J., Cawthorn W. P. & Rosen C. J. Bone marrow adipose tissue: formation, function and regulation. Curr Opin Pharmacol 28, 50–56 (2016). 10.1016/j.coph.2016.03.001 27022859 PMC5351553

[41] Al Saedi A. Age-Related Increases in Marrow Fat Volumes have Regional Impacts on Bone Cell Numbers and Structure. Calcif Tissue Int 107, 126–134 (2020). 10.1007/s00223-020-00700-8 32356017

[42] Justesen J. Adipocyte tissue volume in bone marrow is increased with aging and in patients with osteoporosis. Biogerontology 2, 165–171 (2001). 10.1023/a:1011513223894 11708718

[43] Salmi A., Quacquarelli F., Chauveau C., Clabaut A. & Broux O. An integrative bioinformatics approach to decipher adipocyte-induced transdifferentiation of osteoblast. Genomics 114, 110422 (2022). 10.1016/j.ygeno.2022.110422 35817314

[44] Clabaut A. Adipocyte-induced transdifferentiation of osteoblasts and its potential role in age-related bone loss. PLoS One 16, e0245014 (2021). 10.1371/journal.pone.0245014 33497412 PMC7837466

[45] Gao L., Gong F. Z., Ma L. Y. & Yang J. H. Uncarboxylated osteocalcin promotes osteogenesis and inhibits adipogenesis of mouse bone marrow-derived mesenchymal stem cells via the PKA-AMPK-SIRT1 axis. Exp Ther Med 22, 880 (2021). 10.3892/etm.2021.10312 34194558 PMC8237271

[46] Palhinha L. Leptin Induces Proadipogenic and Proinflammatory Signaling in Adipocytes. Front Endocrinol (Lausanne) 10, 841 (2019). 10.3389/fendo.2019.00841 31920961 PMC6923660

[47] Yue R., Zhou B. O., Shimada I. S., Zhao Z. & Morrison S. J. Leptin Receptor Promotes Adipogenesis and Reduces Osteogenesis by Regulating Mesenchymal Stromal Cells in Adult Bone Marrow. Cell Stem Cell 18, 782–796 (2016). 10.1016/j.stem.2016.02.015 27053299

[48] Xiao Z. & Quarles L. D. Physiological mechanisms and therapeutic potential of bone mechanosensing. Rev Endocr Metab Disord 16, 115–129 (2015). 10.1007/s11154-015-9313-4 26038304 PMC5079521

[49] Li H., Xiao Z., Quarles L. D. & Li W. Osteoporosis: Mechanism, Molecular Target and Current Status on Drug Development. Curr Med Chem 28, 1489–1507 (2021). 10.2174/0929867327666200330142432 32223730 PMC7665836

[50] Reginensi A. Yap- and Cdc42-dependent nephrogenesis and morphogenesis during mouse kidney development. PLoS Genet 9, e1003380 (2013). 10.1371/journal.pgen.1003380 23555292 PMC3605093

[51] Piontek K. B. A functional floxed allele of Pkd1 that can be conditionally inactivated in vivo. J Am Soc Nephrol 15, 3035–3043 (2004). 10.1097/01.ASN.0000144204.01352.86 15579506

[52] Zhang M. Osteoblast-specific knockout of the insulin-like growth factor (IGF) receptor gene reveals an essential role of IGF signaling in bone matrix mineralization. The Journal of biological chemistry 277, 44005–44012 (2002). 12215457 10.1074/jbc.M208265200

